# Machine learning and conventional Cox regression to predict target-lesion revascularization after percutaneous coronary intervention

**DOI:** 10.3389/fcvm.2026.1832496

**Published:** 2026-07-01

**Authors:** Mona El-Faramawi, Marco Busco, Sören Möller, Lisette Okkels Jensen, Jens Flensted Lassen

**Affiliations:** 1 Department of Cardiology, Odense University Hospital, Odense, Denmark; 2 Department of Clinical Research, University of Southern Denmark, Odense, Denmark; 3 Department of Cardiovascular and Pulmonary Sciences, Catholic University of the Sacred Heart, Rome, Italy; 4 Epidemiology, Biostatistics, and Biodemography. Department of Public Health, University of Southern Denmark, Odense, Denmark

**Keywords:** machine learning, percutaneous coronary intervention, prediction model, revascularization, stent failure, target-lesion revascularization

## Abstract

**Introduction:**

Despite advances in procedural and medical treatment, the risk of target-lesion revascularization (TLR) after percutaneous coronary intervention (PCI) persists. This study investigated the use of machine learning (ML)-based Least Absolute Shrinkage and Selection Operator (LASSO) to predict the risk of short- and long-term clinically driven TLR compared with conventional Cox regression with stepwise variable selection.

**Methods:**

The dataset consisted of 24,360 patients with 34,149 *de novo* lesions treated with PCI and stent implantation in Southern Denmark from 2002 to 2022. Forty-eight patient- and procedure-related predictive variables were included. Data were split into 80/20 training and test sets for internal validation. Prediction models for TLR at 0–1 and 1–5 years post-PCI were developed using full Cox regression, Cox with stepwise backward elimination, forward selection, a combination, and ML-based Cox-LASSO. Models were compared using Harrell's C-index. The log-rank test assessed model discrimination between low- and high-risk index lesions of TLR.

**Results:**

Full Cox and stepwise Cox performed equally at 0–1 years (Harrell's C 0.6743). Cox-LASSO provided a minor improvement in predictive performance on the short-term risk of TLR (0.6774). At 1–5 years, stepwise Cox had the best predictive performance (0.6831) and was not outperformed by Cox-LASSO (0.6818). Most identified risk factors for TLR were consistent across conventional Cox models and Cox-LASSO. Survival curves showed separation between high- and low-risk index lesions in all models, as evaluated by the log-rank test.

**Conclusion:**

The ML-based Cox-LASSO model did not improve predictive performance over well-specified conventional Cox regression models for short- and long-term TLR. The models demonstrated intermediate predictive performance and suggest that they can support risk stratification after further validation, but they may not yet be precise enough for definitive bedside decision-making for individual patients.

## Introduction

Ischemic heart disease (IHD) is one of the major threats to public health (1). Contemporary primary and secondary prevention efforts have reduced the incidence and increased the prevalence of IHD (2). Patients undergoing percutaneous coronary intervention (PCI) with stent implantation are at risk of stent failure [in-stent restenosis (ISR) and stent thrombosis (ST)] requiring reintervention (3–5). Drug-eluting stents (DES) have reduced the rate of ISR (6, 7). However, the first-generation DES have been associated with late and very late ST (8, 9). Newer-generation DES with biodegradable polymer for drug release and thinner stent struts have been developed to overcome this problem (10, 11). Both patient- and procedure-related factors may influence the risk of stent failure requiring target-lesion revascularization (TLR) (3). The longer life expectancy among patients with IHD emphasizes the importance of early identification of high-risk lesions to individualize treatment and follow-up at the time of the initial PCI procedure. Conventional statistical approaches, such as Cox regression combined with stepwise variable selection, are widely used for developing clinical prediction models. However, these methods may be limited in their ability to handle large numbers of predictors, identify variable interactions, and automatically select relevant covariates. Machine Learning (ML)-based methods, such as penalized regression models like Least Absolute Shrinkage and Selection Operator (LASSO), may overcome some of these limitations (12, 13). LASSO selects the best model by shrinking some predictor coefficients to zero by penalizing the absolute values of the regression coefficients (12). By shrinking some of the predictor coefficients to zero, LASSO is able to perform automated variable selection and present interpretable models (14).

The aim of this study was to investigate the use of ML-based Cox-LASSO to predict the risk of short- and long-term clinically driven TLR and to stratify patients according to risk, compared to conventional Cox regression with stepwise variable selection, based on information available at the time of the index PCI procedure.

## Methods

### Study design and population

The study was a register-based study based on data extraction from the Western Denmark Heart Registry (WDHR). The WDHR is a multi-center registry containing information on all coronary angiographies (CAGs), PCIs, and cardiac surgeries performed in Western Denmark, covering 3.3 million residents (55% of the Danish population) (15). The internet-based registry mandates the prospective and consecutive registration of all cardiovascular procedures, including detailed information on patient- and procedure-related data. All-comer patients with *de novo* lesions treated with PCI and stent implantation for acute coronary syndrome (ACS) or stable angina pectoris in a native vessel or coronary artery bypass grafting (CABG) at Odense University Hospital from January 1, 2002, to December 31, 2022, were eligible for inclusion. Patients without a Danish personal identifier number were excluded because follow-up was not possible. Subsequent PCI procedures following the initial PCI were categorized as TLR or non-TLR.

### Outcomes

The outcome was the risk of TLR at 0–1 and 1–5 years following the index PCI procedure.

### Definitions

TLR was defined as repeated PCI or CABG of the index lesion in native vessel or CABG due to ISR (more than 50% stenosis within the stent or within 5 mm border proximal or distal to the stent) or definite ST [defined by the American Research Consortium-2 (16)]. Reintervention of index segments and segments adjacent to the index segment, in which ST or ISR had neither been confirmed nor ruled out in the database, were evaluated by manual review of medical records and CAGs. Non-TLR was defined as PCI remote from the previously stented area and any revascularization by CABG without a preceding CAG describing >50% stenosis in the index segment.

### Statistical analyses

Forty-eight patient- and procedure-related predictive variables were included. Missing data were assumed to be missing at random and multiply imputed using predictive mean matching (PMM) with a k-nearest neighbors (with k = 5). The number of imputations was 54, corresponding to the proportion of incomplete cases. Gaussian distribution of data was evaluated visually by histograms and the Shapiro–Wilk test. Continuous variables are presented as mean [± standard deviation (SD)] or median [interquartile range (IQR)] for normally and non-normally distributed data, respectively. Categorical variables are presented as counts with proportions [n (%)]. Follow-up of patients continued until date of death; December 31, 2022; or 5 years of follow-up, whichever came first. Kaplan–Meier cumulative incidence curves were applied to estimate the time-dependent risk of TLR. The full data set was split randomly on lesion-level into a training set (80%) and test set (20%) to allow internal validation on held-out data. Differences in baseline characteristics between training and test set were assessed using the independent t-test, Mann–Whitney U test, or chi-squared-test, depending on data type and distribution. Conventional Cox regression models were full Cox-regression, Cox regression with backward elimination, forward selection, and a combination of both (cut-off *p*-value 0.2 for removal and 0.1 for entry, respectively) developed on the training set. The regression models were adjusted for the patient- and procedure-related variables listed in Table 1 and the year of the index PCI procedure. Additionally, since multiple index lesions could originate from the same patient, within-patient correlation was accounted for by applying cluster-robust sandwich variance estimators at the patient level in the regression analyses. ML-based regularized Cox-LASSO regression was implemented using the official “lasso cox” command in Stata. The penalty parameter (ʎ) was selected using 10-fold cross-validation based on the minimum cross-validation criterion on the training set. The included predictors were the same as for the conventional Cox regression models, including time of the year of the index PCI procedure and within-patient correlation using cluster-robust sandwich variance estimators. Continuous predictors were standardized according to the default Stata implementation. Categorical predictors were included using factor-variable notation and encoded as indicator variables. No interaction terms were included in the model. The selected features by LASSO were extracted and put into a conventional Cox regression model to predict the risk of TLR. Model metrics across conventional Cox regression and ML-based Cox-LASSO were assessed on the held-out test set. Model predictive performance was assessed using Harrell's C statistics (C-index). Accuracy was evaluated using the Brier score. Calibration analyses included calibration plots, slope, and intercept. The log-rank test assessed whether the models effectively distinguished between low- and high-risk index lesions of TLR, using the median predicted risk to create balanced groups and enable standardized comparison across the different prediction models. The proportional hazard assumptions were assessed using the Schoenfeld residual-based test. All analyses were performed using a predefined random seed to guarantee reproducibility and consistency of the results. A *p*-value <0.05 was considered statistically significant. All analyses were performed in Stata/SE 18.0 (StataCorp, College Station, TX).

**Table 1:** Baseline characteristics of the index lesions.

	*N* (% missing)	All lesions (*n* = 34,149)	Training set (*n* = 27,319)	Test set (*n* = 6,830)	*P*-value*
Age (years), median [IQR]	34,149 (0)	67 [58–75]	67 [58–75]	67 [58–75]	0.146
Sex (male), *n* (%)	34,149 (0)	25,291 (74.1)	20,267 (74.2)	5,024 (73.6)	0.289
BMI (kg/m2), median [IQR]	31,059 (9.1)	27.1 [24.8–29.8]	27.1 [24.7–29.8]	27.1 [24.8–29.8]	0.635
Hypertension, *n* (%)	33,325 (2.7)	19,297 (56.5)	15,420 (56.4)	3,877 (56.8)	0.633
Diabetes Mellitus (treated), *n* (%)	32,603 (4.5)	4,836 (14.2)	3,882 (14.2)	954 (14.0)	0.608
Dyslipidemia, *n* (%)	33,307 (2.5)	18,229 (53.4)	14,660 (53.7)	3,569 (52.3)	0.037
Active smoking, *n* (%)	31,544 (7.6)	10,091 (29.6)	8,101 (29.7)	1,990 (29.1)	0.402
Family history of IHD, *n* (%)	30,927 (9.4)	14,542 (42.6)	11,602 (42.5)	2,940 (43.1)	0.389
Previous CABG, *n* (%)	33,789 (1.1)	2,698 (7.9)	2,145 (7.9)	553 (8.1)	0.502
Previous AMI, *n* (%)	33,198 (2.8)	5,515 (16.2)	4,443 (16.3)	1,072 (15.7)	0.254
Previous PCI, *n* (%)	29,888 (12.5)	6,292 (18.4)	5,071 (18.6)	1,221 (17.9)	0.191
Creatinine level (µmol/L), median [IQR]	24,760 (27.5)	87 [76–100]	87 [76–100]	86 [76–100]	0.612
PCI priority, *n* (%)	34,149 (0)				0.138
Acute		11,557 (33.8)	9,217 (33.7)	2,340 (34.3)	
Subacute		10,715 (31.4)	8,531 (31.2)	2,184 (33.0)	
Elective		11,877 (34.8)	9,571 (35.0)	2,306 (33.8)	
Indication for PCI, *n* (%)	34,149 (0)				0.046
STEMI		10,461 (30.6)	8,333 (30.5)	2,128 (31.2)	
NSTEMI/UAP		11,415 (33.4)	9,080 (33.2)	2,335 (34.2)	
Stable angina		12,273 (36.0)	9,906 (36.3)	2,367 (34.7)	
Duration of PCI (minutes), median [IQR]	26,796 (21.5)	23.0 [14.3–39.4]	23.0 [14.3–39.7]	23.6 [14.3–39.0]	0.860
PCI fluoroscopy time (minutes), median [IQR]	34,149 (0)	8.49 [5–15]	8.5 [5–15]	8.5 [4.9–15]	0.801
Contrast during PCI (mL), median [IQR]	34,241 (0)	100 [60–150]	100 [60–150]	100 [60–150]	0.859
No. of diseased vessels, *n* (%)	33,110 (3.0)				0.651
0 vessel disease		123 (0.4)	101 (0.4)	22 (0.3)	
1 vessel disease		16,767 (49.1)	13,442 (49.2)	3,325 (48.7)	
2 vessel disease		11,051 (32.4)	8,841 (32.4)	2,210 (32.4)	
3 vessel disease		6,208 (18.2)	4,935 (18.1)	1,273 (18.6)	
No. of treated lesions, median [IQR]	34,149 (0)	1 [1–2]	1 [1–2]	1 [1–2]	0.373
No. of treated vessels, median [IQR]	34,149 (0)	1 [1–1]	1 [1–1]	1 [1–1]	0.082
No. of used balloons, median [IQR]	34,149 (0)	2 [1–3]	2 [1–3]	2 [1–3]	0.548
No of used stents, median [IQR]	34,149 (0)	1 [1–1]	1 [1–1]	1 [1–1]	0.768
Revascularization (complete), *n* (%)	33,916 (0.7)	28,180 (82.5)	22,517 (82.4)	5,663 (82.9)	0.339
Heparin, *n* (%)	34,115 (0.1)	33,893 (99.3)	27,114 (99.3)	6,779 (99.3)	0.975
Glycoprotein inhibitors, *n* (%)	34,149 (0)	3,657 (10.7)	2,956 (10.8)	701 (10.3)	0.183
Bivalirudin, *n* (%)	34,149 (0)	1,709 (5.0)	1,379 (5.1)	330 (4.8)	0.464
Acetylsalicylic acid, *n* (%)	34,104 (0.1)	33,582 (98.3)	26,870 (98.4)	6,712 (98.3)	0.627
ADP inhibitor, *n* (%)	33,665 (1.4)	33,229 (97.3)	26,585 (97.3)	6,644 (97.3)	0.868
Treated vessel, *n* (%)	34,149 (0)				0.107
Left anterior descending coronary artery		14,207 (41.6)	11,369 (41.6)	2,838 (41.6)	
Circumflex coronary artery		7,208 (21.1)	5,769 (21.1)	1,439 (21.1)	
Right coronary artery		11,381 (33.3)	9,065 (33.2)	2,316 (33.9)	
Left main coronary artery		1,353 (4.0)	1,116 (4.1)	237 (3.5)	
Saphenous vein graft, *n* (%)	30,595 (10.4)	515 (1.5)	417 (1.5)	98 (1.4)	0.579
Type of lesion, *n* (%)	34,149 (0)				0.864
A		7,654 (22.4)	6,129 (22.4)	1,525 (22.3)	
B1		8,971 (26.3)	7,196 (26.3)	1,775 (26.0)	
B2		7,896 (23.1)	6,318 (23.1)	1,578 (23.2)	
C		9,628 (28.2)	7,676 (28.1)	1,952 (28.6)	
Evaluation of lesion, *n* (%)	34,149 (0)				0.309
Eyeballing		33,122 (97.0)	26,490 (97.0)	6,632 (97.1)	
Intravascular imaging		390 (1.1)	306 (1.1)	84 (1.2)	
Physiology		637 (1.9)	523 (1.9)	114 (1.7)	
Chronic total occlusion	30,643 (10.3)	1,017 (3.0)	807 (3.0)	210 (3.1)	0.600
Bifurcation lesion	30,692 (10.1)	2,807 (8.2)	2,284 (8.4)	523 (7.7)	0.058
Calcification	30,964 (9.3)	5,687 (16.7)	4,517 (16.5)	1,170 (17.1)	0.237
Thrombus	31,136 (8.8)	5,054 (14.8)	4,049 (14.8)	1,005 (14.7)	0.824
Direct stenting (yes), *n* (%)	34,142 (0.0)	2,972 (8.1)	2,398 (8.8)	574 (8.4)	0.327
Bifurcation stenting	34,149 (0)	2,078 (6.1)	1,688 (6.2)	390 (5.7)	0.147
Post dilatation	34,149 (0)	5,940 (17.4)	4,695 (17.2)	1,245 (18.2)	0.042
Stent length (mm), median [IQR]	34,149 (0)	18 [15–24]	18 [15–24]	18 [15–24]	0.562
Stent type, *n* (%)	34,149 (0)				0.135
BMS		1,972 (5.8)	1,545 (5.7)	427 (6.3)	
1. generation DES		3,953 (11.6)	3,151 (11.5)	802 (11.7)	
2. generation DES		28,224 (82.7)	22,623 (82.8)	5,601 (82.0)	
Max. balloon pressure (atm), median [IQR]	34,149 (0)	16 [14–18]	16 [14–18]	16 [14–19]	0.407
Balloon diameter (mm), median [IQR]	34,149 (0)	3.4 [3.0–3.8)	3.4 [3.0–3.8]	3.4 [3.0–3.8]	0.611
Reference segment (mm), median IQR]	34,149 (0)	3.2 [2.8–3.5]	3.2 [2.8–3.5]	3.2 [2.8–3.5]	0.769
Min. lumen diameter (mm), median [IQR]	34,145 (0)	0.3 [0.04–0.70]	0.3 [0.0–0.7]	0.3 [0.04–0.70]	0.090
Stenosis before PCI (%), median [IQR]	34,149 (0)	90 [80–99]	90 [80–99]	90 [80–99]	0.111
TIMI flow >2 before PCI, *n* (%)	34,149 (0)	22,761 (66.7)	18,147 (66.4)	4,614 (67.6)	0.077
TIMI flow >2 after PCI, *n* (%)	34,149 (0)	33,475 (98.0)	26,761 (98.0)	6,714 (98.3)	0.067

IQR, interquartile range; BMI, body mass index; IHD, ischemic heart disease; CABG, coronary artery bypass grafting; AMI, acute myocardial infarction; PCI, percutaneous coronary intervention; STEMI, ST-elevation myocardial infarction; NSTEMI, non-ST-elevation myocardial infarction; UAP, unstable angina pectoris; ADP, adenosine diphosphate; BMS, bare-metal stent; DES, drug eluting stent; TIMI, thrombolysis in myocardial infarction.

* *P*-value from independent t-test, Mann–Whitney U test, or chi-squared test comparing training and test sets.

### Ethical considerations

The study was registered and approved by the Region of Southern Denmark (journal no. 23/2002, Acadre no. 23/49957). Ethical approval is not required for register-based research according to the Committee Law §14, as confirmed by the Danish National Committee on Health Research (ref. no. S-20232000-46).

## Results

### Baseline characteristics of the index lesions

**Figure 1:**
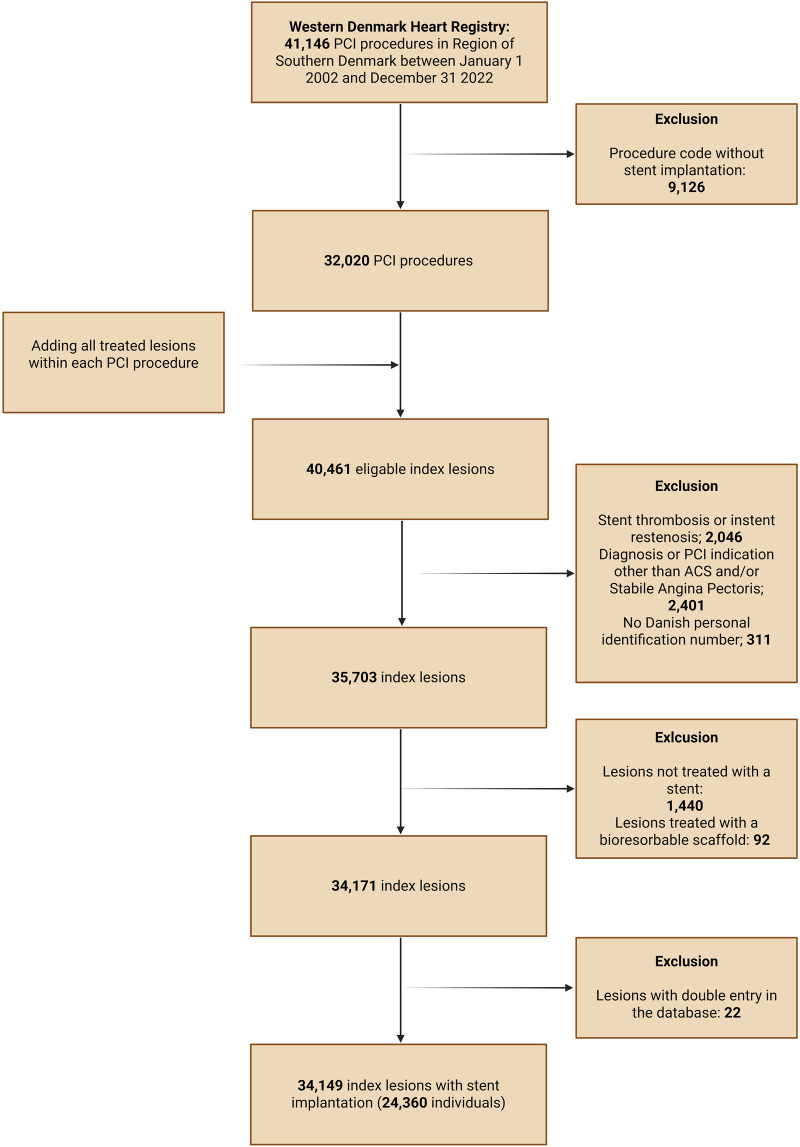
Flowchart of the study population. PCI, Percutaneous Coronary Intervention; ACS, Acute Coronary Syndrome. Created in BioRender. El-Faramawi, M. (2026) https://BioRender.com/1bj3x0c.

A total of 32,020 PCI procedures with stent implantation were performed at Odense University Hospital from January 1, 2002, to December 31, 2022, and the total number of lesions was 40,461. A total of 6,312 lesions were excluded (Figure 1), and the analysis consisted of 24,360 patients with 34,149 index lesions. Most of the index lesions were found in males (74.2%) (Table 1). More than half of the lesions were found in patients with hypertension and dyslipidemia and 13.1% of the patients had diabetes. The left anterior descending coronary artery was the most frequently treated vessel. DES was the predominant stent type, with second-generation DES being the most frequently used. A comparison of the training and test sets revealed that they were similar in distributions, except for a statistically significant difference in the presence of dyslipidemia, indication for PCI, and post dilatation (*p* < 0.05) (Table 1). These differences were considered to reflect random variation.

### Kaplan–Meier cumulative incidence curves

**Figure 2:**
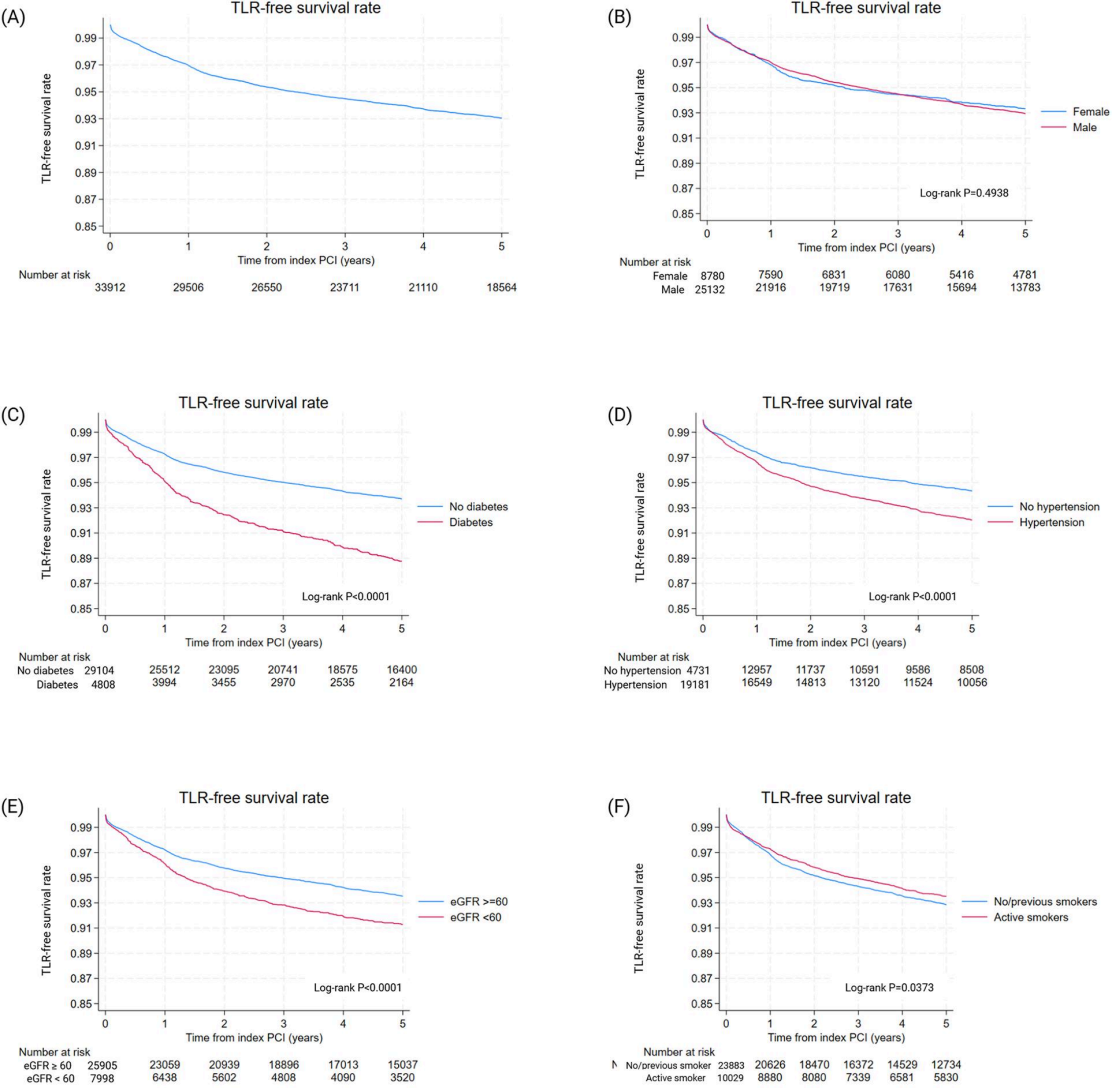
Kaplan–Meier curves for freedom from target-lesion revascularization (TLR) at five-year follow-up. **(A)** overall TLR-free survival rate; **(B)** TLR-free survival rate stratified by sex; **(C)** TLR-free survival rate stratified by diabetes; **(D)** TLR-free survival rate stratified by hypertension; **(E)** TLR-free survival rate stratified by kidney function; **(F)** TLR-free survival rate stratified by smoking status. PCI, percutaneous coronary intervention. Created in BioRender. El-Faramawi, M. (2026) https://BioRender.com/89nldtz.

The total number of events (TLR) in the entire study cohort was 2,497, of which 2,005 appeared within five years after the index PCI procedure (982 events at 0–1 year and 1,023 events at 1–5 years). The median follow-up time was 5 years [IQR 2.4–5.0]. The overall 5-year TLR rate was 7% (Figure 2A). There was no difference in the TLR rate between males and females (Figure 2B). The TLR rate was higher in patients with diabetes mellitus (Figure 2C), in patients with hypertension (Figure 2D), and in patients with an estimated glomerular filtration rate (eGFR) < 60 mL/min/1.73m2 (Figure 2E). The 5-year TLR rate was higher among no/previous smokers (Figure 2F).

### Prediction models

#### 0–1 years

**Figure 3:**
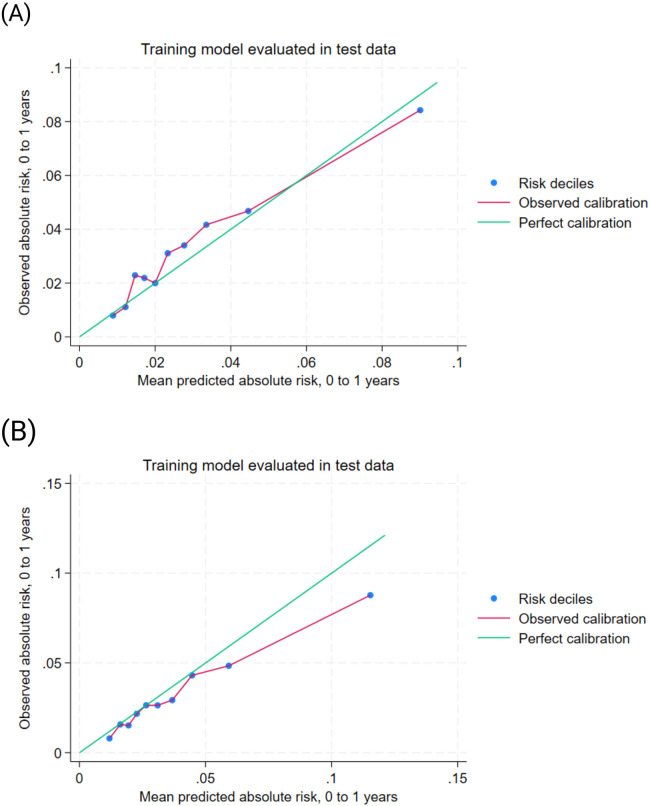
Calibration plots for the best predictive models of 0–1 year TLR risk. **(A)** Calibration plot for Cox regression with backward elimination; **(B)** Calibration plot for Cox-LASSO. Created in BioRender. El-Faramawi, M. (2026) https://BioRender.com/grcva42.

**Figure 4:**
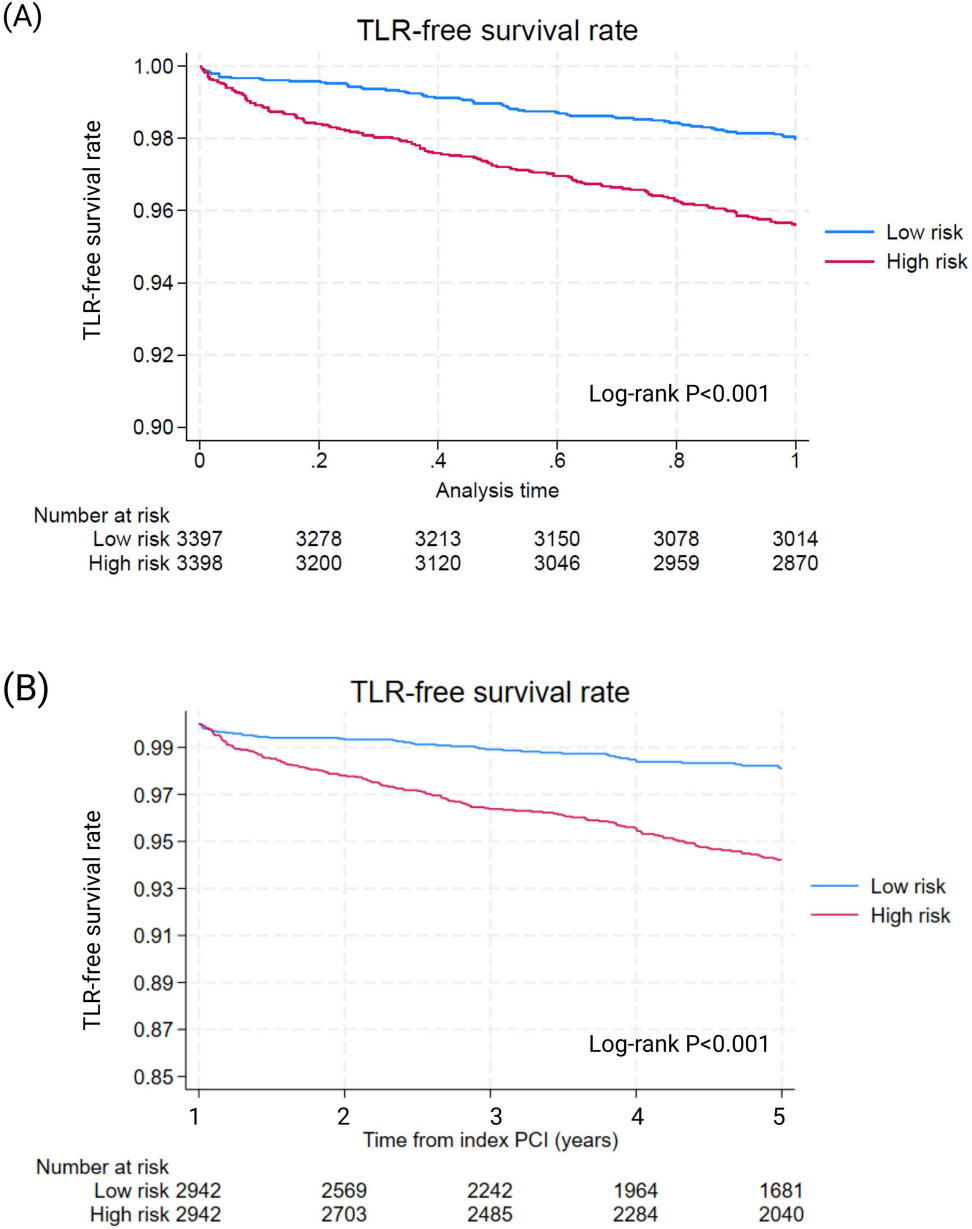
Kaplan–Meier curves for predicted freedom from target-lesion revascularization (TLR) for the best predictive model within each time period. **(A)** TLR-free survival at 0–1 years predicted by Cox regression with backward elimination; **(B)** TLR-free survival at 1–5 years predicted by stepwise Cox with forward selection. PCI = percutaneous coronary intervention. Created in BioRender. El-Faramawi, M. (2026) https://BioRender.com/it58upl.

Full Cox regression, stepwise Cox with backward elimination, and combined stepwise Cox with forward selection and backward elimination achieved similar predictive performance of TLR at 0–1 year (C-index 0.6743) (Table 2). These models demonstrated good accuracy (Brier score <0.1). Stepwise Cox achieved a better calibration compared to the full Cox model (calibration slope 0.9067 in stepwise Cox compared to 0.7467 in full Cox) (Figure 3A showing the calibration plot for Cox with backward elimination). All the conventional models were able to distinguish between high- and low-risk index lesions with a predicted 1-year TLR rate of 4.5% and 2% in the high- and low-risk index lesions, respectively (log-rank, *p* < 0.05) (Figure 4A showing the cumulative incidence curve for the Cox regression with backward elimination). Overall, the models agreed on most identified risk factors (Table 2). Cox-LASSO provided a minor improvement in predictive performance, achieving a C-index of 0.6774 [0.6408–0.7140] compared to the highest C-index of 0.6743 in conventional Cox (Table 2). The accuracy of Cox-LASSO was similar to that of the conventional Cox models, although calibration performance was poorer compared to the conventional stepwise Cox models (Figure 3B showing the calibration plot for Cox-LASSO). Cox-Lasso retained the ability to distinguish between high- and low-risk index lesions.

**Table 2:** Prediction models of TLR at 0–1 years, adjusted for the year of the index PCI procedure.

	Full Cox model		Cox with backward elimination		Cox with forward selection		Combined stepwise Cox^a^		Cox-LASSO	
	HR [95% CI]	*P*-value	HR [95%CI]	*P*-value	HR [95% CI]	*P*-value	HR [95% CI]	*P*-value	HR [95% CI]	*P*-value
Age	0.98 [0.97–0.99]	<0.001	0.98 [0.97–0.99]	<0.001	0.99 [0.98–0.995]	0.002	0.98 [0.97–0.99]	<0.001	0.98 [0.97–0.99]	<0.001
Diabetes	1.57 [1.28–1.94]	<0.001	1.59 [1.30–1.96]	<0.001	1.60 [1.32–1.95]	<0.001	1.60 [1.30–1.96]	<0.001	1.58 [1.29–1.95]	<0.001
Family history of IHD	1.20 [1.01–1.42]	0.037	1.21 [1.02–1.43]	0.029	1.24 [1.05–1.46]	0.012	1.21 [1.02–1.43]	0.029	1.20 [1.02–1.42]	0.032
Previous PCI	1.48 [1.18–1.85]	0.001	1.58 [1.32–1.90]	<0.001	1.62 [1.36–1.94]	<0.001	1.58 [1.32–1.90]	<0.001	1.48 [1.19–1.86]	0.001
Creatinine level	1.001 [1.0002–1.002]	0.014	1.001 [1.0004–1.002]	0.004	1.001 [1.0004–1.002]	0.004	1.001 [1.0004–1.002]	0.004	1.001 [1.0004–1.002]	0.005
Duration of PCI	1.01 [1.002–1.02]	0.010	1.01 [1.001–1.01]	0.032	1.004 [1.0001–1.007]	0.046	1.01 [1.001–1.01]	0.032	1.004 [1.0002–1.01]	0.040
No. of used balloons	1.10 [1.04–1.16]	<0.001	1.09 [1.04–1.15]	0.001	1.10 [1.04–1.16]	<0.001	1.09 [1.04–1.15]	0.001	1.09 [1.04–1.15]	0.001
Complete revascularization	0.60 [0.49–0.74]	<0.001	0.56 [0.46–0.67]	<0.001	0.56 [0.47–0.67]	<0.001	0.56 [0.46–0.67]	<0.001	0.61 [0.49–0.75]	<0.001
Glycoprotein inhibitors	1.56 [1.19–2.04]	0.001	1.55 [1.21–1.99]	<0.001	1.53 [1.20–1.95]	0.001	1.55 [1.21–1.99]	<0.001	1.54 [1.19–2.00]	0.001
Left anterior descending coronary artery	1.25 [1.03–1.52]	0.024	1.20 [1.02–1.41]	0.027	1.20 [1.03–1.41]	0.023	1.20 [1.02–1.41]	0.027	1.24 [1.02–1.50]	0.027
Left main coronary artery	1.99 [1.41–2.83]	<0.001	1.94 [1.42–2.65]	<0.001	2.08 [1.53–2.83]	<0.001	1.94 [1.42–2.65]	<0.001	2.04 [1.47–2.84]	<0.001
Saphenous venous graft	2.14 [1.38–3.32]	0.001	2.32 [1.54–3.38]	<0.001	2.40 [1.61–3.57]	<0.001	2.32 [1.54–3.48]	<0.001	2.15 [1.42–3.28]	<0.001
1. generation DES	0.45 [0.32–0.65]	<0.001	0.44 [0.31–0.62]	<0.001	0.45 [0.32–0.63]	<0.001	0.44 [0.31–0.62]	<0.001	0.45 [0.32–0.64]	<0.001
2. generation DES	0.54 [0.40–0.71]	<0.001	0.52 [0.40–0.69]	<0.001	0.52 [0.40–0.69]	<0.001	0.52 [0.40–0.69]	<0.001	0.53 [0.40–0.70]	<0.001
Maximum balloon pressure	1.04 [1.02–1.07]	0.001	1.05 [1.02–1.07]	0.001	1.04 [1.01–1.07]	0.002	1.05 [1.02–1.07]	0.001	1.04 [1.02–1.07]	0.001
Balloon diameter	0.68 [0.54–0.86]	0.001	0.67 [0.54–0.86]	<0.001	0.76 [0.66–0.87]	<0.001	0.68 [0.53–0.86]	<0.001	0.78 [0.68–0.89]	<0.001
Lesion type B2	1.28 [1.002–1.64]	0.048	-	-	-	-			-	-
Lesion type C	1.39 [1.07–1.81]	0.013	1.35 [1.06–1.71]	0.015	-	-	1.35 [1.06–1.71]	0.015	1.31 [1.04–1.66]	0.024
Calcification	-	-	-	-	1.22 [1.003–1.50]	0.047	-	-	-	-
TIMI >2 after PCI	-	-	-	-	1.92 [1.007–3.66]	0.047	-	-	1.92 [1.002–3.69]	0.049
Model performance metrics										
Harrell's C statistics [95% CI]^b^	0.6743 [0.6385–0.7101]		0.6743 [0.6386–0.7100]		0.6694 [0.6324–0.7064]		0.6743 [0.6386–0.7100]		0.6774 [0.6408–0.7140]	
Brier score [95% CI]^b^	0.0280 [0.024–0.0313]		0.0280 [0.0244–0.0313]		0.0280 [0.0244–0.0313]		0.0280 [0.0244–0.0313]		0.0280 [0.0244–0.0313]	
Calibration [95% CI]										
Slope	0.7467 [0.5705–0.9029]		0.9067 [0.7606–1.0528]		0.9204 [0.7750–1.066]		0.9067 [0.7606–1.0528]		0.7493 [0.6673–0.8312]	
Intercept	0.0057 [−0.0019–0.0133]		0.0057 [0.0003–0.0111]		0.0047 [−0.0007–0.0101]		0.0057 [0.0003–0.0111]		0.0035 [−0.0005–0.0074]	
Log-rank test, *p*-value^c^	<0.0001		<0.0001		<0.0001		<0.0001		<0.0001	

TLR, target lesion revascularization; PCI, percutaneous coronary intervention; HR, hazard ratio; CI, confidence interval; IHD, ischemic heart disease; DES, drug-eluting stent; TIMI, thrombolysis in myocardial infarction.

a Cox regression with forward selection and backward elimination.

b 95% bootstrap confidence interval, estimated using 500 bootstrap repetitions.

c Optimized log-rank test. Performed by dichotomizing lesions in the test set into high- and low-risk groups using the median value derived from the training set.

“-” indicates that the variable was not statistically significant in the model.

#### 1–5 years

**Figure 5:**
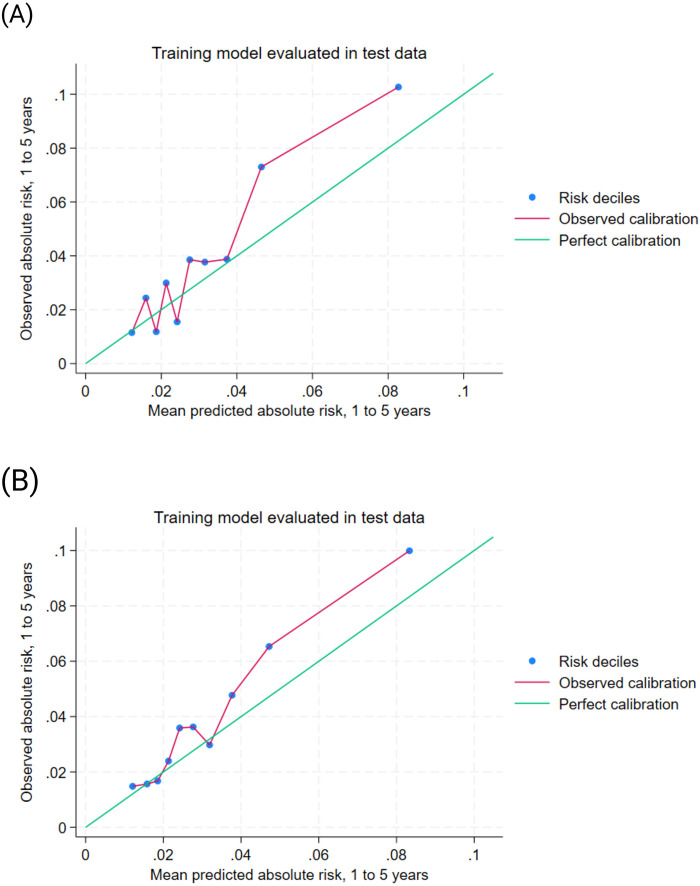
Calibration plots for the best predictive models of 1–5 year TLR risk. **(A)** Calibration plot for Cox regression with forward selection; **(B)** Calibration plot for Cox-LASSO. TLR = target-lesoin revascularization; LASSO, Least Absolute Shrinkage and Selection Operator. Created in BioRender. El-Faramawi, M. (2026) https://BioRender.com/tubl8d3.

Cox with forward selection demonstrated the best model metrics in predicting the risk of TLR at 1–5 years following the index PCI [C-index 0.6831 (0.6478–0.7235)] (Table 3). The accuracy was 0.0309 [0.0109–0.0166], the calibration slope was 1.3415 [1.0057–1.6773] (Figure 5A), and the model was able to distinguish between high- and low-risk index lesions (TLR rate of 6% and 2%, respectively) (Figure 4B). The majority of the identified independent risk factors in stepwise Cox with forward variable selection were consistent across all conventional Cox models. The performance of the other conventional models was also intermediate with C-index ranging from 0.6794 to 0.6818 (Table 3). Cox-LASSO did not outperform stepwise Cox (C-index 0.6818), and it did not identify additional independent risk factors compared to conventional Cox regression models. However, it did achieve a better calibration [calibration slope 1.2544 (1.0520–1.4569)] (Figure 5B).

**Table 3:** Prediction models of TLR at 1–5 years, adjusted for the year of the index PCI procedure.

	Full Cox model		Cox with backward elimination		Cox with forward selection		Combined stepwise Cox^a^		Cox-LASSO	
	HR [95% CI]	*P*-value	HR [95%CI]	*P*-value	HR [95% CI]	*P*-value	HR [95% CI]	*P*-value	HR [95% CI]	*P*-value
Age	0.98 [0.97–0.98]	<0.001	0.98 [0.97–0.98]	<0.001	0.98 [0.97–0.98]	<0.001	0.98 [0.97–0.98]	<0.001	0.98 [0.97–0.98]	<0.001
Hypertension	1.27 [1.06–1.52]	0.011	1.26 [1.05–1.51]	0.012	1.28 [1.06–1.53]	0.009	1.26 [1.05–1.51]	0.012	1.26 [1.05–1.51]	0.013
Diabetes	1.39 [1.14–1.70]	0.001	1.38 [1.13–1.68]	0.001	1.39 [1.14–1.69]	0.001	1.38 [1.13–1.68]	0.001	1.37 [1.12–1.67]	0.002
Previous PCI	1.72 [1.37–2.17]	<0.001	1.67 [1.40–1.99]	<0.001	1.70 [1.43–2.02]	<0.001	1.67 [1.40–1.99]	<0.001	1.65 [1.37–1.97]	<0.001
Creatinine level	1.003 [1.002–1.003]	<0.001	1.002 [1.002–1.003]	<0.001	1.003 [1.002–1.003]	<0.001	1.002 [1.002–1.003]	<0.001	1.003 [1.002–1.003]	<0.001
Saphenous venous graft	3.25 [2.17–4.86]	<0.001	3.35 [2.38–4.71]	<0.001	3.46 [2.47–4.84]	<0.001	3.35 [2.38–4.71]	<0.001	3.28 [2.22–4.85]	<0.001
Lesion type C	1.37 [1.08–1.75]	0.011	1.36 [1.08–1.70]	0.009	1.26 [1.07–1.48]	0.005	1.36 [1.08–1.70]	0.009	1.38 [1.10–1.73]	0.006
Maximum balloon pressure	1.04 [1.01–1.07]	0.003	1.04 [1.02–1.07]	0.001	1.04 [1.02–1.07]	0.002	1.04 [1.02–1.07]	0.001	1.04 [1.01–1.07]	0.002
Reference segment	0.77 [0.59–0.99]	0.043	0.79 [0.70–0.90]	<0.001	0.78 [0.69–0.89]	<0.001	0.79 [0.70–0.90]	<0.001	0.79 [0.69–0.91]	<0.001
Left main coronary artery	-	-	1.52 [1.05–2.20]	0.027	-	-	1.52 [1.05–2.20]	0.027	-	-
PCI fluoroscopy time	-	-	1.01 [1.0001–1.01]	0.048	1.01 [1.001–1.01]	0.029	1.006 [1.0001–1.01]	0.048	-	-
Model performance metrics										
Harrell's C statistics [95% CI]^b^	0.6818 [0.6424–0.7211]		0.6794 [0.6401–0.7187]		0.6831 [0.6478–0.7235]		0.6794 [0.6401–0.7187]		0.6818 [0.6422–0.7214]	
Brier score [95% CI]^b^	0.0137 [0.0110–0.0166]		0.0137 [0.0109–0.0166]		0.0137 [0.0109–0.0166]		0.0137 [0.0109–0.0166]		0.0309 [0.0271–0.0347]	
Calibration [95% CI]										
Slope	1.1997 [0.9547–1.4447]		1.3616 [1.1038–1.1620]		1.3415 [1.0057–1.6773]		1.3616 [1.1038–1.6195]		1.2544 [1.0520–1.4569]	
Intercept	0.0021 [−0.0067–0.0110]		−0.0024 [−0.0116–0.0068]		−0.0043 [−0.0168–0.0083]		−0.0024 [−0.0116–0.0068]		−0.0015 [−0.0091–0.0061]	
Log-rank test, *p*-value^c^	<0.0001		<0.0001		<0.0001		<0.0001		<0.0001	

TLR, target lesion revascularization; PCI, percutaneous coronary intervention; HR, hazard ratio; CI, confidence interval; IHD, ischemic heart disease; DES, drug-eluting stent; TIMI, thrombolysis in myocardial infarction.

a Cox regression with forward selection and backward elimination.

b 95% bootstrap confidence interval, estimated using 500 bootstrap repetitions.

c Optimized log-rank test. Performed by dichotomizing lesions in the test set into high- and low-risk groups using the median value derived from the training set.

“-” indicates that the variable was not statistically significant in the model.

The LASSO-selected variables that were included in the short- and long-term Cox-LASSO regressions are listed in Supplementary Table 1 along with the coefficients for each selected variable.

### Predictors of TLR

Age, diabetes mellitus, a history of previous PCI, and creatinine level were identified as patient-related independent predictors for short- and long-term TLR risk across all conventional Cox models and the Cox-LASSO models (Tables 2, 3). Older age was associated with a lower risk of TLR, while diabetes mellitus, a previous history of PCI, and higher creatinine level were associated with an increased risk of TLR after the index PCI. At 0–1 years, a larger number of procedure-related variables were identified as independent predictors for TLR, including the use of first- and second-generation DES being associated with a lower short-term risk.

### Sensitivity analyses

Among the index lesions, 21,876 (64.1%) were indicated by ACS. The number of events (TLR) among ACS lesions was 1,137, with 474 appearing within one year and 449 appearing at 1–5 years. Overall, sensitivity analyses restricted to ACS index lesions demonstrated model metrics similar to those observed for conventional Cox regression and Cox-LASSO in the overall analyses (Supplementary Table 2). At 0–1 year, the best predictive performance was achieved by Cox with backward elimination and the combined Cox model [0.6790 (0.6346–0.7235)]. The calibration slopes for those models were close to 1, although the corresponding confidence intervals were relatively wide. Cox-LASSO did not outperform stepwise Cox. However, both the conventional models and the Cox-LASSO model were able to statistically distinguish between high- and low-risk index lesions.

At 1–5 years, Cox with backward elimination and the combined Cox model achieved the best predictive performance [0.6487 (0.5870–0.7103)], with good accuracy and calibration slopes (Supplementary Table 2). Cox-LASSO did not outperform stepwise Cox, and it demonstrated poorer calibration despite similar accuracy. All models retained the ability to stratify the study population into high- and low-risk groups.

## Discussion

This study made a comparison of the predictive performance of the ML-based Cox-LASSO with conventional Cox with stepwise variable selection in predicting the risk of short- and long-term clinically driven TLR after PCI with stent implantation. Overall, conventional Cox and Cox-LASSO demonstrated intermediate predictive performance. Full Cox and stepwise Cox performed equally well at 0–1 years and almost similar to Cox-LASSO. At 1–5 years, stepwise Cox demonstrated the best predictive performance and was not outperformed by Cox-LASSO. All models within each time period were able to stratify the study population in high- and low-risk groups.

Using a large dataset of > 34,000 index lesions with stent implantation with 48 patient- and procedure-related predictive variables, four conventional Cox regression prediction models with stepwise variable selection and Cox-LASSO were derived. Cox and stepwise Cox regressions are widely used in scientific time-to-event analyses to predict a clinical outcome. Developing a prediction model requires a thorough evaluation of which variables to include and how many to keep the model stable, interpretable, and simple for clinical practice. Additionally, one must actively check for collinearity and interactions between variables based on existing evidence and clinical knowledge. A large number of variables and unknown multi-collinearity and interactions may cause the models to become unstable. In the current study, full Cox and stepwise Cox performed equally in the short-term risk analyses, while stepwise Cox was best in the long-term risk analyses. Given the large dataset, the number of variables included in the models is unlikely to pose concerns regarding the ratio between observations and variables. However, conventional Cox models do not highlight the importance of the variables included, which makes it difficult to exclude variables in order to improve model stability. Furthermore, the lack of events within variable categories may increase over time, especially causing instability in analyses conducted several years after the index PCI procedure. This could affect the predictive performance of the risk of TLR over an even longer period, e.g. > 5 years post-PCI. However, the ability of conventional statistics to present extreme hazard ratio values in unbalanced and highly predictive covariates, also known as “monotone likelihood” (17), may help identify statistically problematic variables to improve model predictive performance. Overall, this highlights the importance of variable selection in conventional statistical prediction models, as the choice of the predictors is closely tied to the outcome definition and time horizon. Consequently, predictor selection is influenced by researcher-driven decisions, which may introduce subjectivity and limit the objectivity of the resulting models. This limitation may be overcome by using ML-based models, that have the potential to improve cardiovascular research (18). Regular automated stepwise variable selection methods are commonly used in medical applications to include only the most significant predictors in the models (12). The ML-based penalized Cox regression (Cox-LASSO) for model selection and shrinkage in Cox proportional hazard models may be more accurate than stepwise selection (12, 13).

Ojeda et al. (19) have conducted a similar study that compares different approaches to computing Cox proportional hazards models. These approaches included a crude (full) model, backward elimination, and cross-validated LASSO. The study examined the ability to predict non-fatal myocardial infarction and cardiovascular mortality among patients with coronary artery disease in rare event scenarios with a median follow-up time of 5.7 years. They found that LASSO outperformed Cox and stepwise Cox regression. The results of a stepwise Cox regression process are affected by the predefined threshold (*p*-value) of the analyses. Ojeda et al. (19) used a lower *p*-value for elimination than the current study. A lower threshold results in more variables being eliminated because fewer predictors will meet the stricter significance criterion required to remain in the model. Conversely, a higher threshold may retain too many variables, including those with a weak or non-existent association with the outcomes. This can lead to overfitting with reduced generalizability and potentially worse performance on new data. In other words, stepwise Cox is not fully automated and depends on pre-analytic decisions. No literature explicitly defining optimal cut-off values for thresholds was identified. The *p*-value thresholds selected for removal and entry in the current study were chosen to strike between underfitting and overfitting, as recommended in the Stata manual (20). Furthermore, the data-driven nature of stepwise selection makes it suitable for exploratory model building but limits its validity for statistical inference on individual predictors. LASSO, on the other hand, being effective in reducing model complexity with only the most relevant variables, is widely used in ML (21, 22). Despite the large sample size and number of variables, which are advantageous for ML-based survival modeling using LASSO-penalized Cox regression, it performed similarly to conventional Cox at predicting short-term and long-term risk of TLR. However, Cox-LASSO could simplify conventional statistical models by eliminating variables that are frequently included but may contribute little to overall predictive performance when considered alongside other variables. In prediction models, it is preferable to have a model with high predictive performance and a minimal number of variables, as this facilitates easier implementation in clinical practice. That is an important distinction compared to association studies, in which the primary focus is on estimating associations between exposure and outcome and therefore requires careful adjustment for all relevant confounders (23). The study population was stratified into high- and low-risk groups based on median predicted values. This pragmatic approach was used for exploratory risk stratification across the models, since the study focused on comparative model performance. Determination of clinically actionable threshold and assessment of clinical utility warrant further investigation. An external validation would be needed as the next step, after which the best-performing model could be selected for further independent refinement and optimization. Additionally, calibration analyses showed slopes below one in the short-term prediction of TLR, suggesting a potential degree of overfitting, whereas the calibration slopes were above one in the long-term prediction of TLR, indicating possible underfitting. These findings further support the need for external validation.

The models were developed to identify high-risk patients that would benefit from closer follow-up after PCI. Predicting the risk of both ST and ISR as TLR increases statistical power by capturing more events. On the other hand, the identified risk factors within each time period may be driven by one type of stent failure more than the other, which could reduce the specificity of targeting particular subtypes of clinically driven stent failures. ST, which has a more severe clinical presentation and a higher mortality rate than ISR (3, 24), is expected to be more prevalent in the short-term analyses (3). Separate models for ST and ISR might achieve higher predictive performance, as the models might find it easier to identify predictors separately for each outcome. However, separate models would reduce the number of events per model, potentially compromising model stability in conventional statistics. To enable a fair comparison between conventional statistical models and the penalized Cox regression, the number of events within each time period was maximized by using TLR, thereby reducing constraints related to the ratio between observations, events, and covariates in traditional regression-based analyses.

Distinguishing between short- and long-term TLR risk after PCI could enable risk-guided clinical management and facilitate individualized post-PCI treatment (such as the length of dual antiplatelet therapy) and follow-up, as early TLR may predominantly be driven by procedure- or device-related risk factors, whereas late TLR could be more likely influenced by disease progression and patient-related characteristics (25, 26). The current study was a prediction study. The identified predictors may inform future association studies of the relevant risk factors of short- and long-term TLR risk. The LASSO-penalized Cox regression model applied in the current study is considered interpretable, as predictor effects are represented directly by regression coefficients in a simplified model (13). The more complex the ML-based algorithms, the more careful one should be when drawing conclusions about possible clinical associations, which is one of the major limitations of using advanced ML in health care research. However, Cox-LASSO may be considered as a relatively simple ML-based extension of conventional regression methods, although it remains partly constrained by some of the assumptions underlying Cox regression. Assessment of the proportional hazard assumptions in the current study suggested violations for some predictors. However, the aim of the present study was to explore risk modeling and methodological comparison of conventional Cox with the ML-based Cox-LASSO rather than inference. The conventional Cox framework was therefore retained as an interpretable benchmark model against the more flexible ML-based Cox-LASSO, taking into account that the observed hazard ratio (HR) will constitute time-averaged HR for those predictors with deviations from the proportional hazard assumptions. This suggests exploration of more advanced ML-based algorithms that are fully data-driven and without assumptions regarding the predictors and outcome.

### Strengths

The large dataset was essential for exploring the possibilities and limitations of conventional statistical methods and the ML-based penalized Cox-regression. The mandatory registration and data entry into the WDHR database reduce the risk of selection bias at the time of inclusion (15). This increases the generalizability of research studies based on WDHR. Additionally, data were split into a training and test set to allow for internal validation on unseen data to reduce the risk of overfitting. The event rate was maximized by manual evaluation whether a reintervention in segments adjacent to the index segment was related to the previously inserted stent reducing the risk of missing clinically driven stent failures.

### Limitations

Limitations of this study include the lack of migration data. Patients who received an index PCI in the Region of Southern Denmark but were residents of, or moved to, another region of Denmark or abroad were not eligible for follow-up. However, this number is expected to be low. Another limitation was the amount of missing data, which were addressed using PMM k-NN multiple imputation. There was no external validation of the developed models. Such validation will be necessary before considering any clinical implementation of prediction models.

## Conclusion

The ML-based Cox-LASSO model did not improve predictive performance over well-specified conventional Cox regression models for short- and long-term risk of TLR. The models demonstrated intermediate predictive performance and suggest that they can support risk stratification after further validation, but they may not yet be precise enough for definitive bedside decision-making for individual patients.

## Data Availability

The data underlying this article will be shared on reasonable request to the corresponding author, provided that the necessary regulatory and data access approvals are obtained.
