# Supplementary material for: Machine learning and conventional Cox regression to predict target-lesion revascularization after percutaneous coronary intervention
Source: Front Cardiovasc Med. 2026 Jul 1;13:1832496. doi: 10.3389/fcvm.2026.1832496 (PMC13368786; doi:10.3389/fcvm.2026.1832496)
Supplement: Supplementary file 1 [file Table1.docx]

**Supplemental Table 1. Selected variables by Cox-LASSO.**

| Supplemental Table 1. | | | | | |
| --- | --- | --- | --- | --- | --- |
| 0-1 years | **Coefficient [95% CI]** | **P-value** | **1-5 years** | **Coefficient [95% CI]** | **P-value** |
| Age | -0.02 [-0.03-(-0.01)] | **0.000** | **Age** | -0.02 [-0.03-(-0.02)] | 0.000 |
| Sex, male | -0.16 [-0.36-0.03] | 0.099 | **Hypertension** | 0.23 [0.05-0.41] | 0.013 |
| BMI | -0.02 [-0.04-0.00] | 0.073 | **Diabetes mellitus** | 0.32 [0.12-0.51] | 0.002 |
| Diabetes mellitus | 0.46 [0.25-0.67] | **0.000** | **Dyslipidemia** | 0.14 [-0.06-0.33] | 0.177 |
| Dyslipidemia | 0.14 [-0.05-0.34] | 0.144 | **Family history of IHD** | 0.11 [-0.05-0.27] | 0.165 |
| Active smoking | -0.16 [-0.35-0.03] | 0.091 | **Previous CABG** | -0.01 [-0.28-0.27] | 0.952 |
| Family history of IHD | 0.18 [0.02-0.35] | **0.032** | **Previous PCI** | 0.50 [0.32-0.68] | 0.000 |
| Previous AMI | 0.06 [-0.18-0.30] | 0.616 | **Creatinine level** | 0.003 [0.002-0.003] | 0.000 |
| Previous PCI | 0.40 [0.17-0.62] | **0.001** | **Index PCI indication** |  |  |
| Creatinine level | 0.001 [0.0003-0.002] | **0.005** | STEMI | -0.10 [-0.34-0.14] | 0.396 |
| Index PCI indication |  |  | NSTEMI/UAP | -0.12 [-0.30-0.07] | 0.227 |
| STEMI | 0.07 [-0.17-0.30] | 0.582 | **Fluoroscopy time at index PCI** | 0.01 [-0.002-0.01] | 0.206 |
| NSTEMI | 0.12 [-0.08-0.31] | 0.248 | **No. of diseased vessels** |  |  |
| Duration of PCI | 0.004 [0.0002-0.01] | **0.040** | 1 vessel disease | -0.03 [-1.15-1.09] | 0.954 |
| No. of diseased vessels |  |  | 2 vessel disease | -0.08 [-1.21-1.04] | 0.883 |
| 1 vessel disease | -0.23 [-1.72-1.26] | 0.762 | 3 vessel disease | 0.04 [-1.10-1.18] | 0.943 |
| 2 vessel disease | -0.18 [-1.68-1.32] | 0.814 | **No. of treated vessels** | -0.10 [-0.29-0.11] | 0.383 |
| 3 vessel disease | -0.05 [-1.55-1.46] | 0.949 | **No. of used balloons** | 0.02 [-0.04-0.08] | 0.513 |
| No. of treated lesions | 0.08 [-0.07-0.24] | 0.269 | **Revascularization** | -0.12 [-0.34-0.10] | 0.292 |
| No. of treated vessels | -0.25 [-0.50-0.01] | 0.059 | **ADP-antagonist** | 0.41 [-0.28-1.09] | 0.241 |
| No. of used balloons | 0.09 [0.04-0.14] | **0.001** | **Treated vessel*** |  |  |
| Revascularization | -0.50 [0.71-(-0.29)] | **0.000** | Circumflex coronary artery | -0.03 [-0.24-0.18] | 0.762 |
| Glycoprotein inhibitors | 0.43 [0.17-0.69] | **0.001** | Left anterior descending coronary artery | -0.08 [-0.25-0.10] | 0.410 |
| Acetylsalicylic acid | -0.27 [-0.79-0.26] | 0.319 | Left main coronary artery | 0.36 [-0.03-0.75] | 0.074 |
| Treated vessel^a^ |  |  | **Saphenous venous graft** | 1.19 [0.80-1.58] | 0.000 |
| Circumflex coronary artery | 0.06 [-0.16-0.29] | 0.581 | **Lesion type**** |  |  |
| Left anterior descending coronary artery | 0.22 [0.02-0.41] | **0.027** | B1 | 0.15 [-0.06-0.37] | 0.160 |
| Left main coronary artery | 0.71 [0.39-1.04] | **0.000** | B2 | 0.17 [-0.05-0.40] | 0.127 |
| Saphenous venous graft | 0.78 [0.35-1.19] | **0.000** | C | 0.32 [0.09-0.55] | 0.006 |
| Lesion type^b^ |  |  | **Evaluation of index PCI^c^** |  |  |
| B1 | 0.15 [-0.09-0.39] | 0.222 | Intravascular imaging | 0.02 [-0.78-0.82] | 0.966 |
| B2 | 0.21 [-0.03-0.45] | 0.090 | Physiology | -0.31—0.97-0.36] | 0.364 |
| C | 0.27 [0.03-0.51] | **0.024** | **Chronical total occlusion** | -0.37 [-0.77-0.04] | 0.074 |
| Calcification | 0.16 [-0.05-0.36] | 0.131 | **Bifurcation lesion** | -0.24 [-0.55-0.08] | 0.145 |
| Bifurcation stenting | 0.12 [-0.17-0.40] | 0.429 | **Thrombus** | -0.09 [-0.34-0.16] | 0.475 |
| Stent type*** |  |  | **Direct stenting** | 0.21 [-0.05-0.47] | 0.111 |
| 1. gen DES | -0.79 [-1.14-(-0.44)] | **0.000** | **Post dilatation** | -0.08 [-0.32-0.17] | 0.526 |
| 2. gen DES | -0.63 [-0.91-(-0.36)] | **0.000** | **Stent length** | 0.00 [0.00-0.01] | 0.352 |
| Maximum balloon pressure | 0.04 [0.02-0.07] | **0.001** | **Stent type^d^** |  |  |
| Balloon diameter | -0.25 [-0.39-0.11] | **0.000** | 1. gen DES | 0.34 [-0.04-0.72] | 0.082 |
| TIMI flow >2 after PCI | 0.65 [0.002-1.30] | **0.049** | 2. gen DES | 0.20 [-0.15-0.56] | 0.261 |
|  |  |  | **Maximum balloon pressure** | 0.04 [0.1-0.06] | **0.002** |
|  |  |  | **Reference segment** | -0.23 [-0.37-(-0.10)] | **0.001** |

LASSO = Least Absolute Shrinkage and Selection Operator; BMI = body mass index; IHD = ischemic heart disease; AMI = acute myocardial infarction; PCI = percutaneous coronary intervention; TIMI = thrombolysis in myocardial infarction; CABG = coronary artery bypass grafting; ADP-antagonist = adenosine diphosphate antagonist.

^a^Right coronary artery (RCA) is the reference the reference group.

^b^Type A lesion is the reference group.

^c^Eyeballing is the reference group.

^d^Bare-metal stent (BMS) is the reference group.
