# Supplementary material for: Machine learning and conventional Cox regression to predict target-lesion revascularization after percutaneous coronary intervention
Source: Front Cardiovasc Med. 2026 Jul 1;13:1832496. doi: 10.3389/fcvm.2026.1832496 (PMC13368786; doi:10.3389/fcvm.2026.1832496)
Supplement: Supplementary file 2 [file Table2.docx]

**Supplemental Table 2. Predictive performance among ACS patients**

| Supplemental Table 2. | | | | | |
| --- | --- | --- | --- | --- | --- |
|  | Full cox model | Cox with backward elimination | Cox with forward selection | Combined stepwise cox^a^ | Cox-LASSO |
| 0-1 year |  |  |  |  |  |
| Harrell’s C statistics [95% CI]^b^ | 0.6777  [0.6333-0.7210] | 0.6790  [0.6346-0.7235] | 0.6701  [0.6251-0.7151] | 0.6790  [0.6346-0.7235] | 0.6766  [0.6319-0.7214] |
| Brier score [95% CI]^b^ | 0.0283  [0.0238-0.0328] | 0.0283  0.0238-0.0328] | 0.0283  [0.0238-0.0328] | 0.0283  [0.0238-0.0328] | 0.0283  [0.0238-0.0328] |
| Calibration [95% CI]^b^ |  |  |  |  |  |
| Slope | 0.3422  [0.1934-0.4911] | 1.1177  [0.6741-1.5610] | 0.9800  [0.6786-1.2815] | 1.1176  [0.6741-1.5610] | 0.4834  [0.2679-0.6989] |
| Intercept | 0.0093  [-0.0040-0.0027] | 0.0093  -0.0032 [0.0217] | 0.0107  [.0018-0.0196] | 0.0093  [-0.0032-0.0217] | 0.0103  [-0.0029-0.0235] |
| Log-rank test, p-value^c^ | **<0.001** | **<0.001** | **<0.001** | **<0.001** | **<0.001** |
| 1-5 years |  |  |  |  |  |
| Harrell’s C statistics [95% CI]^b^ | 0.6320  [0.4757-0.7882] | 0.6487  [0.5870-0.7103] | 0.6345  [0.5737-0.6952] | 0.6487  [0.5870-0.7103] | 0.6366  [0.4774-0.7957] |
| Brier score [95% CI]^b^ | 0.0247  [0.0203-0.0291] | 0.0247  [0.0203-0.0291] | 0.0247  [0.0203-0.0291] | 0.0247  [0.0203-0.0291] | 0.0247  [0.0203-0.0291] |
| Calibration [95% CI]^b^ |  |  |  |  |  |
| Slope | 1.4347  [0.9857-1.8838] | 1.2167  [0.9709-1.4629] | 1.5184  [1.1378-1.8990] | 1.2169  [0.9709-1.4629] | 0.2403  [0.1490-0.3315] |
| Intercept | 0.0059  [-0.0037-0.0155] | 0.0042  [-0.0024-0.0108] | 0.0016  [-0.0074-0.0105] | 0.0042  [-0.0024-0.0108] | 0.0069  [-0.0053-0.0192] |
| Log-rank test, p-value^c^ | **0.006** | **<0.001** | **0.001** | **<0.001** | **0.003** |

ACS = acute coronary syndrome; LASSO = least absolute shrinkage and selection operator.

^a^Cox regression with forward selection and backward elimination.

^b^95% bootstrap confidence interval, estimated using 500 bootstrap repetitions.

^c^Optimized log-rank test. Performed by dichotomizing lesions in the test set into high- and low-risk groups using the median value derived from the training set.
